# Canine Models of Inherited Musculoskeletal and Neurodegenerative Diseases

**DOI:** 10.3389/fvets.2020.00080

**Published:** 2020-03-11

**Authors:** Brett D. Story, Matthew E. Miller, Allison M. Bradbury, Emily D. Million, Dongsheng Duan, Toloo Taghian, Dominik Faissler, Deborah Fernau, Sidney J. Beecy, Heather L. Gray-Edwards

**Affiliations:** ^1^Auburn University College of Veterinary Medicine, Auburn, AL, United States; ^2^University of Florida College of Veterinary Medicine, Gainesville, FL, United States; ^3^Department of Clinical Sciences and Advanced Medicine, University of Pennsylvania, Philadelphia, PA, United States; ^4^Department of Molecular Microbiology and Immunology, School of Medicine, University of Missouri, Columbia, MO, United States; ^5^Department of Biomedical, Biological and Chemical Engineering, College of Engineering, University of Missouri, Columbia, MO, United States; ^6^Department of Biomedical Sciences, College of Veterinary Medicine, University of Missouri, Columbia, MO, United States; ^7^Department of Neurology, School of Medicine, University of Missouri, Columbia, MO, United States; ^8^Horae Gene Therapy Center, University of Massachusetts Medical School, Worcester, MA, United States; ^9^Cummings School of Veterinary Medicine at Tufts University, North Grafton, MA, United States; ^10^Department of Radiology, University of Massachusetts Medical School, Worcester, MA, United States

**Keywords:** canine, translational, large animal model, gene therapy, neurodegeneration

## Abstract

Mouse models of human disease remain the bread and butter of modern biology and therapeutic discovery. Nonetheless, more often than not mouse models do not reproduce the pathophysiology of the human conditions they are designed to mimic. Naturally occurring large animal models have predominantly been found in companion animals or livestock because of their emotional or economic value to modern society and, unlike mice, often recapitulate the human disease state. In particular, numerous models have been discovered in dogs and have a fundamental role in bridging proof of concept studies in mice to human clinical trials. The present article is a review that highlights current canine models of human diseases, including Alzheimer's disease, degenerative myelopathy, neuronal ceroid lipofuscinosis, globoid cell leukodystrophy, Duchenne muscular dystrophy, mucopolysaccharidosis, and fucosidosis. The goal of the review is to discuss canine and human neurodegenerative pathophysiologic similarities, introduce the animal models, and shed light on the ability of canine models to facilitate current and future treatment trials.

## Introduction

The utility of canine models of neurodegenerative diseases has increased over the last decade. Each year, children are born with rare and devastating diseases, and characterizing underlying pathologies remains a constant challenge. This is often attributed to a lack of appropriate animal models. Dog models are bridging the gap in scientific studies because they share similar pathophysiology to their human counterparts. The strong recapitulation across species is pivotal for development of future therapies for human diseases. This is best shown by the numerous lysosomal storage diseases (LSDs) that have been described in dogs and these models have and will continue to play a vital role in development of safe and efficacious treatments for humans (1).

The canine genome has strong similarities to the human, and the increased size and complexity of the dog brain makes it a suitable large animal model of neurologic disease (1, 2). As models, dogs are of a size suitable for surgical guidance and permit clinical evaluation over time. A dog's weight, metabolism, and pharmacokinetics are closer to a human's than is a rodent's, allowing therapeutics to be tested for efficacy and toxicity in larger doses before moving to human trials. Coupled with the fact that they are relatively outbred, share a similar environment, and develop many similar neurologic and immunologic diseases, they represent an ideal animal model to study disease pathogenesis and novel therapeutics. For example, the immunological similarities in X-linked severe combined immunodeficiency (XSCID), where the immunologic defect in dogs with XSCID helped lead to the discovery of the gene responsible for the human disease (3). Although mouse models are invaluable for the initial assessment of gene therapy, use of dog models with these natural diseases is an important step in moving toward human clinical trials.

## Alzheimer's and Canine Cognitive Dysfunction

### Alzheimer's Disease in Humans

Alzheimer's disease (AD) is the 6th leading cause of death in the United States with a worldwide prevalence of 44 million people in 2015 (4, 5). AD is a slowly progressive disease that is insidious in onset with initial clinical signs of recent short-term memory loss and sparing of long-term memory (5, 6). Personality changes, irritability, aphasia, apraxia, and agnosia amongst a multitude of other neurodegenerative clinical signs, usually manifest after several years (6). AD patients can live on average 8–10 years after diagnosis, with the disease lasting up to 20 years (7). A diagnosis of AD comprises a reliable history, physical and neurological examinations, routine laboratory examinations, advanced imaging, and neuropathology.

There are two recognized forms of AD: familial or early-onset, and sporadic or late-onset AD (LOAD). Most affected individuals (>95%) suffer from sporadic or (LOAD), a multifactorial disease where environmental factors and genetic predispositions contribute to pathology (4, 8). A small minority (around 5%) of patients suffer from familial or early-onset AD due to genetic mutations in following genes: amyloid precursor protein (APP), presenilin 1, and presenilin 2 (9–11). The hallmark neuropathological features of AD are extracellular accumulation amyloid plaques (amyloid-β peptide) and intracellular accumulation of neurofibrillary tangles. There are multiple hypotheses of AD pathogenesis which are beyond the scope of this review.

Risk factors associated with LOAD can be either genetic or environmental. The strong genetic component of LOAD is due to apolipoprotein E (APOE) which has three allelic forms: APOE-2, -3, and -4 (4, 6). The APOE4 allele is the most important genetic risk factor and appears to enhance amyloid-β peptide aggregation and/or decrease its clearance (6, 12). Non-genetic environmental causes include type 2 diabetes, obesity, hypercholesterolemia, hyperhomocysteinemia, and hypertension (8, 13–19). There is no cure for AD and treatments are largely supportive. Most treatments consist of pharmacotherapy (cholinesterase inhibitors, NMDA antagonists) and psychosocial therapy (environmental manipulation, family support, prevention of additional medical comorbidities) (5, 20–24).

### Canine Cognitive Dysfunction

Canine Cognitive Dysfunction (CCD) is a common neurodegenerative disorder in geriatric canine patients. The exact pathogenesis remains undetermined but is believed to be due to the neurotoxic effects of beta-amyloid protein accumulation in the brain. The prevalence of CCD in dogs over the age of 8 ranges from 14 to 60% (25–28). Dogs naturally accumulate amyloid-β peptide in their brains, with an identical amino acid sequence to humans and similar amounts of deposition associated with cognitive impairment (29–33). While neurofibrillary tangles have not reported in CCD dogs, a pre-tangle pathology has been suggested (31, 34–37). Further, dogs do not appear to show as significant cognitive defects as AD patients (e.g., loss of ability to eat) suggesting that CCD progression is more comparable to early stage AD (38).

In one long term observational study the most common clinical signs in dogs with CCD included: altered sleep wake cycles, altered interactions, disorientation, and anxiety in ~50% of CCD dogs (39). The DISHA acronym is commonly used to summarize clinical signs with CCD and includes disorientation, altered interactions, sleep wake cycle disturbances, house soiling, and changes in activity level (40). Concurrent medications, other medical problems, natural sleep disturbances, and general anxiety must be ruled out before an accurate diagnosis can be reached. CCD dogs have age-related changes on MRI (interthalamic adhesion thickness ≤ 5 mm) that are consistent with, but not specific for CCD (41). Another study found that interthalamic adhesion thickness, interthalamic adhesion thickness/brain height ratio, and lateral ventricle to brain height ratio were all significantly lower in dogs with signs of cognitive dysfunction than in dogs with no cognitive function. These metrics were validated for use in quantifying brain atrophy in patients (42). Like AD, CCD cannot be cured. The goals of treatment are to slow disease progression and improve clinical signs. CCD is often managed with environmental enrichment, nutritional therapy (Hill's b/d, Purina One Vibrant Maturity), oral L-deprenyl (selegiline), and supplementation with S-adenosylmethionine.

### Therapeutic Testing in the Canine Model

Transformative results have not been noted in CCD dogs treated with any therapy tested to date. A diet rich in antioxidants like Vitamins A and E, fatty acids like DHA and EPA, and mitochondrial protectants like lipoic acid and L-carnitine, have been shown to slow cognitive decline in dogs (43–47). A diet rich in medium-chain triglycerides (>6.5%) has been shown to improve cognitive function in dogs with CCD as quantified by 6 out of 6 metrics after 90 days, compared with 4 out 6 metrics for control diets (48). Selegiline therapy has shown some benefit in dogs with CCD when dosed at 0.5–1.0 mg/kg orally for 60 days, with 67.8% of dogs showing improvement in their activity levels or sleep/wake cycles, and 77.8% showing less disorientation or unfamiliarity with family members (49). Oral S-adenosylmethionine (SAMe) tosylate supplementation has also been evaluated in CCD dogs over 8 years old at 18 mg/kg orally (50). By various behavioral and locomotive metrics, 57.1–59.5% of dogs treated with SAMe improved after 8 weeks, compared with 9.0–21.4% of dogs in the placebo group. Other proposed therapies that have not yet been adequately studied include anti-inflammatories (51, 52), phytochemicals to prevent oxidation in the brain (53), drugs that enhance cerebral perfusion (52, 54), and drugs that reduce anxiety or normalize the sleep-wake cycle (53).

### Impacts on Patients With Disease

CCD closely resembles the early stages of AD in people, and it is largely accepted that early intervention is better, therefore there is great utility using the CCD dog as an animal model for testing emerging AD therapies. Clinical trials of novel therapeutics for AD have not been tested in CCD dogs to our knowledge, and this is likely due to the complexities associated with conducting client owned animal clinical trials and/or a lack of a comprehensive natural history study of CCD. Dogs with CCD can achieve a full lifespan (39), especially if managed with a combination of dietary modifications (45) and environmental enrichment (55), in contrast to AD patients who live an average of 8–10 years after diagnosis. Therefore, objective measures that determine efficacy will need to be based on the natural history of CCD, including cognitive, behavioral, locomotive and/or image-based measures. A comparative study has potential to reveal new avenues of therapy for AD to minimize or slow progression to the lethal stages of the disease.

## ALS and Degenerative Myelopathy

### Amyotrophic Lateral Sclerosis in Humans

Amyotrophic lateral sclerosis (ALS) is progressively paralytic fatal motor neuron neurodegenerative disorder described by degeneration of upper and lower motor neurons in the brain and spinal cord (56, 57). Amyotrophic refers to muscle atrophy due to denervation from neuron degeneration, while lateral sclerosis refers to the hardened nature of the posterior and lateral spinal cord tracts. ALS is recognized in two forms: around 10% of cases are familial (FALS), usually inherited as an autosomal dominant trait (58–61), and 90% of cases are sporadic with no genetic inheritance pattern. Clinical signs usually present around 50–65 years of age and manifest as twitching, cramping, and muscle weakness which can progress to dyspnea and dysphagia in advanced stages of the disease (62–65).

In general, it has been presumed that ALS is a disease influenced by equally important genetic and environmental factors. Environmental factors with possible correlations to ALS have been studied extensively. Cigarette smoking, military service, exposure to heavy metals, pesticide exposure, and trauma are all risk factors under investigation for association with ALS (66–69). Although sporadic ALS accounts for the majority of cases, genetic causes have been known to play a role (70). FALS involves mutations at specific genetic loci inherited in an autosomal dominant pattern (71–73). FALS and sporadic ALS are clinically and pathologically identical and show genetic similarities (56, 72). Beginning with the discovery of the SOD1 gene in 1993, at least 25 genes have now been associated with familial ALS, sporadic ALS, or both (57, 71, 74–76). Five of these genes account for the majority of familial cases: C9orf72, SOD1, FUS, TARDBP, and ANG (77–83). Mutations in SOD1 and C9orf72 cause familial ALS most commonly with varying rates, with 50–60% of familial ALS patients show mutations arising from the genes identified to date (84).

The main pathologic observation in patients with ALS is upper and lower motor neuron death in the motor cortex and spinal cord, with corticospinal axonal degeneration leading to thinning and sclerosis of the lateral portions of the spinal cord (57). In response to degeneration of motor neurons, neuroinflammatory processes lead to proliferation in microglia, astrocytes, and oligodendrocytes (85, 86). A pathologic common feature in familial and sporadic ALS is the presence of cytoplasmic protein inclusion bodies, mainly but not exclusively in motor neurons (57). In many cases of ALS, the main component of these inclusion bodies is caused by aggregation of proteins encoded by TARDBP, SOD1 or FUS (56, 57, 87). There are four recognized phenotypic expressions of ALS: Limb-onset ALS which shows upper motor neuron (UMN) and lower motor neuron (LMN) signs of the limbs; bulbar onset ALS, described by disruption of cranial nerves 9–12 resulting in speech and swallowing difficulties with limb deterioration in later disease stage; primary lateral sclerosis (PLS) which is described by pure UMN involvement of the corticospinal and corticopontine motor neurons; and lastly, progressive muscular atrophy (PMA) where lower motor involvement dominates (56, 57). On average there is a delay of 13–18 months from onset of clinical signs to diagnosis (88). ALS is a clinical diagnosis of exclusion from other diseases that cause progressive UMN and LMN dysfunction, such as multifocal motor neuropathy with conduction block (57, 89, 90). Ultimately, ALS invariably proves fatal with few FDA approved drugs. The main cause of mortality in patients with ALS is respiratory failure (91).

### Degenerative Myelopathy in Canines

Canine degenerative myelopathy (DM) presents clinically similar to primary lateral sclerosis in ALS patients (92). In dogs, DM is most common in older, large breed dogs, with German Shepherds being classically overrepresented. More recently, DM has been found in many other breeds, such as Pembroke Welsh Corgis (PWCs) and Boxers (92–94). The age of onset of neurologic signs is usually >5 years of age, with large breed dogs having a mean of 9 years and PWCs having a mean of 11 years of age. No sex predilection is apparent. DM refers to older dogs with diffuse axonal necrosis in the ventral and lateral funiculi of the thoracolumbar spinal cord segments with secondary demyelination and astrogliosis (95). DM presents as a slowly progressive, non-painful T3-L3 myelopathy that will over time progress cranially. The disease usually presents as UMN pelvic limb paresis with general proprioceptive ataxia. Over time, animals develop UMN bladder dysfunction, progressive paraparesis, thoracic limb weakness, lower motor neuron weakness, and dyspnea. Compared to larger breed dogs, small breed dogs may have slower disease progression (92).

It is important for veterinary clinicians to recognize that DM is a diagnosis of exclusion with a definitive diagnosis based on post-mortem histopathologic examination of spinal cord segments. Some differential diagnoses for DM include intervertebral disc disease, neoplasia, degenerative lumbosacral syndrome, and degenerative joint diseases, such as hip dysplasia, osteoarthritis, or cranial cruciate ligament ruptures (96). A presumptive diagnosis is often made based on clinical signs, signalment, and absence of compressive myelopathy on MRI or myelography. CSF analysis is often performed to rule out other causes, such as meningitis. CSF may have evidence of albuminocytological dissociation without elevations in cell count. However, a study on PWCs in 2007 showed no cytologic or protein abnormalities on CSF analysis (97).

The underlying cause of degenerative myelopathy is currently unknown. Metabolic, auto-immune, nutritional, and oxidative pathologies have all been suggested. Genome-wide association in PWCs DNA lead to the discovery of the highest concentration of single nucleotide polymorphism in the region of chromosome 31, which contains the SOD1 gene (93). The authors determined a G to A transition in exon 2 in PWCs with DM with all five dog breeds (PWCs, GSDs, Boxers, Chesapeake Bay Retrievers, and Rhodesian Ridgebacks) having confirmed DM being homozygous for the SOD1 mutation. DM DNA testing is available through the University of Missouri. The test determines whether patients are heterozygous, homozygous, or lack the mutation but cannot confirm whether a patient has DM. Dogs homozygous for the SOD1 mutation are at risk for developing DM and the test is commonly used in breeders determined to reduce incidence of DM (92). DM carries a poor long-term prognosis. No therapy has been effective, but most clinicians treat patients with a variety of antioxidants and supplement B12 vitamins. Physical rehabilitation may also help to slow the progression of the disease (98).

### Therapeutic Testing in the Canine Model

No treatment significantly increases survival time for canine patients with DM. However, therapeutic benefits of specific gene silencing of the superoxide dismutase (*SOD1*) gene is being explored as a novel treatment for dogs diagnosed with the disease (99). In dogs with naturally occurring DM, experimental treatment will include adeno-associated virus AAV-Rh10-miR anti-*SOD1* injected into the subarachnoid space to explore whether ambulatory function will persist longer compared to control dogs. Other metrics of interest between experimental and control groups in this study include neurological scoring, measurement of inguinal circumference of the hindlegs, stepping pattern measured by force plate analysis, CBC and chemistry profiles, and degree of *SOD1* silencing based on CSF collection. Although vitamin B12 deficiency has been documented in 50% of German Shepherd dogs with DM, anecdotal evidence suggests that supplementation of B12 is not shown to attenuate the progression of the disease. In people, B12 supplementation has shown success in the treatment of degenerative cervical myelopathy, suggesting the clinically similar diseases have dissimilar etiologies (100).

The ER foldase called protein disulphide isomerase (PDI) has been identified as a reliable indicator of both DM in dogs and ALS in people, as PDI is significantly upregulated in examined tissues from both disease processes compared with controls. Furthermore, given co-localization of PDI and SOD1 accumulation on histopathology, it is thought that PDI may function as a cellular defense mechanism against the misfolding of SOD1. For this reason, PDI may represent a new target for developing therapies against DM and ALS (101).

### Impact on Patients With This Disease

Gene therapy is already being explored extensively as a therapy for people with ALS (102). Although clinical trials for novel therapeutics in ALS patients are showing promising results, it remains useful to test new therapies in dogs with DM as an animal model for ALS, given significant overlap in the pathophysiology, especially the shared importance of the *SOD1* gene in both diseases. Setbacks in human trials may necessitate further DM canine studies to explore either minor modifications in protocol or new techniques entirely.

In light of the substantially lower proportion of people who develop ALS as a result of an SOD1 mutation compared with dogs who develop DM as a result of this mutation, there is some debate as to whether these two conditions are truly the same. Regardless, both DM and ALS result in up-regulated expression of the endoplasmic reticulum stress marker GRP78/BiP (103). Therefore, even if the pathophysiology of DM mimics only a fraction of ALS cases (namely, those mediated by SOD1 mutations), DM may remain a useful animal model for ALS insofar as it could afford us insights into the interplay between the unfolded protein response (UPR) of microglia and astrocytes and the spinal cord inflammation seen in the two diseases.

## Neuronal Ceroid Lipofuscinosis

### Neuronal Ceroid Lipofuscinosis in Humans

The neuronal ceroid lipofuscinoses (NCL) are a group of inherited lysosomal storage disorders described in humans and multiple animal species. With one exception, the NCLs exhibit an autosomal recessive mode of inheritance (104). The incidence of NCL is the United States is 1:12,500 (105) with 14 different forms of NCL being described (CLN1-14) (106). Although the function of many of the NCL genes remain unknown, it is apparent that they are vital for homeostasis of cerebral neurons (107). To illustrate, the products of CLN1, CLN2, and CLN10 are lysosomal storage enzymes while products of CLN3, CLN6, and CLN8 are transmembrane proteins with currently unknown functions (104). Dementia is the main clinical sign see in adult forms of NCL while most childhood forms have varying clinical features that include progressive loss of eyesight, mental and motor deterioration, seizures, and premature death (107).

Although clinical signs are variable, the NCLs share similar pathological characteristics: accumulation of an autoflorescent, periodic acid-Schiff (PAS)- and Sudan black B-positive granules in the lysosomes of neurons and other extraneuronal tissues (104). Cerebral and cerebellar atrophy, increased ventricular size, decreased gyri thickness with loss of nerve cell bodies, loss of myelin, astrocytosis, microgliosis, and ceroid lipopigment accumulation may be observed histologically (108). Other common pathologies noticed are optic and retinal atrophy as well as accumulation of lipopigments in lymphocytes and monocytes (108). NCL can overlap clinically with many other neurodegenerative diseases. As such, one must demonstrate accumulation of autoflorescent ceroid material in nervous tissue to classify a disease as possible NCL (109).

### Neuronal Ceroid Lipofuscinosis in Canines

All NCL genes currently identified in animals are homologs of established NCL genes in humans, meaning that in contrast to AD/CCD or ALS/DM, NCL is agreed to be the same disease happening in two species (104). The first clinical description of NCL in canines was recognized in the 1950's by Dr. Nils Koppang in English Setters as a generalized metabolic disease with accumulation of lipopigments in almost every tissue. This accumulation was most pronounced in the central, but not peripheral, nervous system. Accumulation of intraneuronal ceroid in canines is present at the time of birth, prior to onset of clinical signs. Canine patients suspected for NCL typically show at least four of the following progressively neurologic clinical signs: loss of vision, changes in behavior, losses of learned behavior, cerebellar ataxia, tremors, cognitive and motor decline, sleep cycle disruptions, and convulsions.

A strong advantage of the canine model is the more than 50-year natural history of canine NCL. Since then, NCLs have been identified in at least 18 different canine breeds (110) with various other breeds having reported NCL like symptoms without genetic confirmation. While the earlier descriptions of canine NCL focused largely on phenotype and histopathology (111, 112), later studies utilized new technologies to define the genetic mutations responsible for the disease, including CLN8 mutations in English Setters (113), CLN5 in Border Collies (114), and CLN12 in Australian Cattle Dogs (115). The identification of these mutations, along with the clinical descriptions, allowed parallels to be made between human and canine disease. For example, the CLN8 mutation in English Setters with NCL disease resembles juvenile human NCL (113). The long history of investigation into the various types of canine NCL makes it an extremely valuable large animal model.

Although canines and humans share many phenotypic similarities, there are some differences in presentation between canine and human NCLs of similar genetic origin. For example, English Setters do not show the intense retinal decline that is a hallmark of the human disease (112) and Border Collies show marked behavioral problems that are not as striking in their human counterparts (114). Additionally, different NCL mutations may be difficult to distinguish based on presentation in canines, because symptoms are similar across mutations (114). Canine NCL patients display diffuse cerebral and cerebellar atrophy with an enlarged ventricular system on MRI examination. Since the clinical signs of NCLs mimic other lysosomal storage diseases, definitive diagnosis is based on presence of a mutation in one of the established NCL genes (116). Fortunately, dogs suspected of suffering from NCL can be tested easily for any of the known mutations from a blood sample or cheek swab (117). Like humans, dogs show differences in disease onset, lesion distribution, and chemical storage material. This is consistent with the view that human NCL may not be chemically or genetically homogenous (118).

Spontaneous and induced rodent models of NCL have their own advantages but are also limited by their phenotypic differences from human disease, small size, and limited cognition (113). Spontaneous NCL has been described in a broad range of large animals, including cows, horses, and sheep (114), but canine models have unique qualities that set them apart. Not only do canine genetic mutations mirror human disease, but the symptoms and clinical signs of NCL, such as aggression, agitation, hallucinations, loss of learned behaviors, and blindness are similar between humans and canines (114).

### Therapeutic Testing in the Canine Model

Because of its early identification, canine NCL models have been used for clinical testing since the 1990s (119). Early treatments tested in canine NCL models include bone marrow transplantation (119) and dietary therapies (120). More recently, the miniature longhaired Dachshund model of CLN2-related NCL has been used to test the effectiveness of enzyme replacement therapies (121, 122). Recombinant human tripeptidyl peptidase-1 (rhTPP1) was infused into the CSF of affected Dachshunds every other week and showed a dose-dependent delay in the onset of neurological deficits and an increased life span, but no effect on retinal degeneration (122). Additionally, TPP1 adeno associated viral gene therapy (AAV) was also tested in CLN2 Dachshunds, with a one-time intracerebroventricular injection resulting in delayed disease progression and increased lifespan, although retinal deterioration persisted (123). Both treatments revealed potential benefit for human CLN2 patients, elucidated the need for simultaneous retinal targeting, and led to initiation of human clinical trials (122, 123).

### Impacts on Patients With This Disease

Current treatments of NCL in human patients include Cerliponase alfa (recombinant human TPP1 proenzyme) for CLN2 disease, involving ventricular infusions every other week (124). Recent studies in dog models of NCL suggest that multiple intracranial treatments of rhTPP1 could offer dramatic improvements (122), and a recombinant PPT1 proenzyme treatment has proved promising in CLN1 disease mouse models (124). The one-time ventricular infusion of AAV-TPP1 investigated in CLN2 Dachshunds (123) is in phase I/II clinical trials (124). Other gene therapy solutions have entered clinical trial for CLN6 and CLN3 disease, initially tested in mice (124). Other emerging treatments include immunomodulatory agents and stem cell therapy.

## Globoid Cell Leukodystrophy

### Globoid Cell Leukodystrophy in Humans

Globoid cell leukodystrophy (GLD), also known as Krabbe disease (KD), is a fatal lysosomal storage disease that affects both the central and peripheral nervous systems. GLD is caused by an autosomal recessive mutation on human chromosome 14 (125) affecting the β-galactocerebrosidase gene (GALC), resulting in enzymatic deficiency of galactosylceramidase. Deficiency in this enzyme results in decreased degradation of two lipid substrates: galactosylceramide and psychosine. Clinical onset is divided into four groups: infantile, late infantile, juvenile, and adult. The infantile phenotype comprises 95% of cases with onset of clinical signs within the first 6 months of life and death by 2 years of age (126, 127). With infantile onset, the first initial clinical signs include hypersensitivity, stiffness of limbs, hyperirritability, and fever of unknown origin. As the disease progresses, regression of psychomotor functions and convulsions present. Toward the end of the disease, infants are decerebrate, unaware of their surroundings, and blind. Clinical signs in the later onset forms are more variable with the adult onset patients potentially living a normal life span.

Unlike most LSDs, the secondary substrate, psychosine, is more pronounced than the primary substrate (Gal-cer) and believed to be the major cause of pathogenesis and cytotoxicity (128–134). The hallmarks of GLD include rapid loss of myelination and oligodendrocytes, reactive astrogliosis, and the presence of unique globoid cells. Due to loss of galactosylceramidase, Gal-cer cannot be broken down and has been shown to elicit infiltration of macrophages into the brain (135, 136). The presence of macrophages, coupled with inability of Gal-cer breakdown owed galactosylceramidase deficiency, leads to macrophage phagocytosis of undegradable Gal-cer and characteristic multinucleated Periodic-acid-Schiff positive globoid cells (128).

Psy (galactosylsphingosine) is a cytotoxic lipid that accumulates in the nervous system. Although closely related to Gal-cer, psy causes fatal hemorrhagic infarcts (133) in the brain and is highly cytotoxic (137). The “psychosine hypothesis” has been demonstrated and has survived over the years. The hypothesis states that not only the primary substrate, but also the secondary cytotoxic substrate, psy, cannot be degraded and its accumulation causes loss of the myelin-forming cells (138, 139). Classically, histopathologic presence of multinucleated macrophages engulfed with undegradable Gal-cer was the original means of diagnosis (140, 141). Since then, enzymatic and genetic assays have been developed and are valuable for a prenatal diagnosis (128, 129, 142, 143). Unfortunately, there is no cure for GLD. Currently, the only treatment available is hematopoietic stem cell transplantation, which is only indicated for pre-symptomatic patients.

### Globoid Cell Leukodystrophy in Canines

In addition to humans, globoid cell leukodystrophy (GLD) has been documented in several other species, including dogs, mice, monkeys, and sheep. In dogs, GLD has been reported in Cairn terriers, West Highland Terriers, Australian Kelpies, Beagles, Poodles, and mixed breeds (144–150). As with NCL, GLD is thought to be the same disease occurring in different species, maximizing the usefulness of canine models in developing therapies for humans with GLD. Canine GLD was first described in West Highland terriers and Cairn terriers in 1966 (151). Since its discovery, significant advancements in understanding canine GLD have been made. Affected dogs are homozygous for an autosomal recessive missense mutation and typically present with ataxia or paresis of the pelvic limbs, conscious proprioceptive deficits, and dysmetria. Spinal reflexes may be normal early in disease and affected as the disease progresses. Onset of clinical signs occur by 4–6 weeks of age with a survival time of 15.7 ± 4.8 weeks (149). Analysis of leukocyte β-galactocerebrosidase levels leads to a definitive diagnosis (147, 152).

Histopathology of the brain shows diffuse, massive accumulations of previously described globoid cells, as well as extensive gliosis and perivascular lymphocytic cuffing confined to the white matter with sparing of gray matter (153). Demyelination of all areas of the brain, auditory system, and peripheral nerves is present in canine patients (149). Psychosine is elevated in tissues and CSF of GLD dogs (154) increasing significantly over time and may be a potential biomarker of disease (149). Although MRI and electrodiagnostics cannot accurately achieve a definitive diagnosis, they aid to increase the index of suspicion of GLD in canine patients and warrant blood sample testing for the defective gene.

### Therapeutic Testing in the Canine Model

Combination intravenous and intracerebroventricular delivery of AAVrh10 expressing canine GALC was evaluated in the GLD dog to target peripheral and central nervous system disease, respectively. This treatment approach increased GALC enzyme activity, reduced psychosine, and improved myelination in both the CNS and PNS. Clinically, AAVrh10-cGALC delayed the onset of clinical signs, improved nerve conduction velocity, but did not correct hearing deficits. Gene therapy treated dogs lived up to 43 weeks of age (154). In order to improve targeting of the CNS, an intrathecal delivery approach with AAV9 is currently being investigated in the GLD dog. Pre-symptomatic high dose AAV9 gene therapy resulted in significant improvement on CNS and PNS disease, while lowering the dose and delaying the treatment was less effective (unpublished data).

### Impact on Patients With This Disease

Hematopoietic stem cell transplantation (HSCT) is the only treatment for presymptomatic infantile GLD; however, outcomes of transplanted patients remain poor and there are no therapeutic options for symptomatic patients. Moreover, HSCT does not treat the PNS disease associated with GLD (155). Data in animal models suggest that a one-time injection of AAV expressing GALC could offer dramatic improvements above HSCT without the associated risk of morbidity and mortality.

## Duchenne Muscular Dystrophy

### Duchenne Muscular Dystrophy in Humans

Duchenne muscular dystrophy (DMD) is an invariably fatal neuromuscular X-linked recessive disorder caused by mutations in the dystrophin gene, affecting 250,000 people worldwide and one in every 5,000 male infants. The dystrophin gene is one of the largest genes known in nature and encodes for the cytoskeletal muscle protein dystrophin. Due to its large size, the dystrophin gene is susceptible to mutations. Approximately 60% of dystrophin mutations are large deletions and duplications. The remaining 40% are point mutations or small frameshift rearrangements. These mutations result in reading frame shift and premature termination of protein translation. Dystrophin and its naturally existing isoforms are significant because they are present in nearly every cell in the body. The exact function of dystrophin remains unclear, but roles may include myocyte protection, cell motility, cell division, myocyte contractility, vesicle and organelle movements, cell signaling, and maintaining cell junctions and shape. In the muscle, dystrophin and its associated protein complex are important in stabilizing the plasma membrane by connecting the extracellular matrix and cytoskeleton. Without dystrophin, muscle contraction (especially eccentric contraction) leads to sarcolemma damage, muscle degeneration and necrosis. Historically, death occurs due to respiratory illness secondary to diaphragmatic compromise, but with better ventilation systems and antibiotic treatment, fewer deaths have been attributed to respiratory disease, with more patients dying from cardiac involvement.

Clinical signs are not apparent at birth but begin to appear around 2–4 years of age (156). Abnormal gait and motor delay are the symptoms seen most frequently. Proximal limb weakness in the pelvic limbs occurs prior to thoracic limb involvement and often leads to wheelchair dependence in patients by 11–12 years of age (157). As the disease progresses, chronic respiratory insufficiency and dilated cardiomyopathy, due to cardiac fibrosis, arise and are the major causes of mortality. The exact mechanism by which the loss of dystrophin leads to muscle disease remains unclear, however different hypotheses have been proposed, such as mechanical damage, loss of calcium homeostasis, oxidative stress and failed regeneration (158).

As a consequence of muscle cell membrane damage, muscle enzymes, such as creatine kinase, leak out to circulation. Hence, a major serum chemistry finding in patients prior to 5 years of age is massive increase in serum creatine kinase, at least 10–20 times the upper reference interval. (159). Histological examinations of muscle biopsies show degenerating necrotic muscle fibers with macrophage invasion. Molecular testing is the main means of diagnosis of DMD with a multiplex ligation-dependent probe amplification and targeted high-density oligonucleotide comparative genomic hybridization microarray, both being available commercially. Management of DMD is often tailored to the cardiac and respiratory complications. Early cardiac treatment with ACE inhibitors and beta blockers is often implemented in addition to spirometry, polysomnography, nocturnal non-invasive ventilation, and fulltime ventilation for respiratory decompensation (159). The only approved gold standard pharmacological therapy is corticosteroids, such as prednisone/prednisolone and Deflazacort, although various additional treatment options, including gene therapy, utrophin modulation, aminoglycoside antibiotics, antisense oligonucleotides, and stem-cell therapy are under investigation (160, 161).

### Canine X-Linked Muscular Dystrophy

The classic animal model for DMD is the mdx mouse. Although many advancements in understanding pathogenesis and treatments have been made utilizing the mdx mouse model, these mice express a milder disease phenotype with less severe muscle changes (162). The most common and well-studied model for DMD research is the golden retriever muscular dystrophy (GRMD) model. Variations of the disease have since been documented in other breeds, including Labradoodles, Labradors, Samoyed, Brittney Spaniels, Irish terrier, Miniature Schnauzers, Pembroke Welsh Corgi, German Shorthaired Pointer, and Rat Terriers [reviewed in (163)]. As with the storage diseases, muscular dystrophy in people and in dogs are thought to be the same disease occurring in different species.

DMD in dogs is known as canine X-linked muscular dystrophy (CXMD). In GRMD, there is a point mutation that disrupts splicing causing the dystrophin reading frame to be terminated. Clinical signs of affected dogs are in general much more severe than in mdx mice. CXMD dogs may display muscle atrophy, slow growth, alterations in gait, ptyalism, dysphagia, plantigrade stance, lumbar kyphosis and lordosis, exercise intolerance, inactivity, paradoxical muscular hypertrophy, cardiomyopathy, and congestive heart failure. Spinal reflexes may be intact early in the course of the disease and diminish as the disease progresses.

Like humans, marked elevations in serum muscle enzymes (such as creatine kinase, aspartate aminotransferase, and alanine aminotransferase) are often present due to muscle fiber damage. Within the first week of life dystrophic dogs may be identified by serum CK levels which may be up to 300 times normal (164). A definitive diagnosis is made utilizing muscle biopsies. Skeletal muscle biopsies of affected individuals show severe muscle necrosis, fiber hypercontraction, and regeneration of almost all skeletal muscles (164). The necrosis is accompanied by macrophages, fibrosis, and lipid accumulation with immunohistochemistry staining revealing a lack of dystrophin in the muscle cell membranes (95). Some CXMD breeds (such as GRMD and Welsh Corgi) can also be diagnosed with genomic DNA PCR (165, 166).

### Therapeutic Testing in the Canine Model and Impact on Patients With This Disease

Glucocorticoids are the most commonly used drug in DMD patients. Several groups have tested prednisone in affected dogs (167–169). These studies revealed some beneficial effects, such as improvement in gait, increase of the extensor muscle force, and elevation of the integrin level. However, the treatment also resulted in side effects, such as reduction of flexor muscle force, and more severe muscle pathology, especially calcification. CXMD dogs have also been used to test other anti-inflammatory drugs, such as the NF-κB pathway inhibitor the nemo binding domain (NBD) peptide (170), edasalonexent, and CAT-1041(171). These studies suggest that anti-inflammatory therapy can reduce muscle disease in the canine model. Unfortunately, long-term intravenous administration of NBD was found to induce untoward infusion reactions even in normal dogs. In addition to anti-inflammatory drugs, CXMD dogs have also been used to test calpain inhibitor (172), proteasome inhibitor (173), bradykinin (174, 175), and laminin-111 protein therapy (176). Proteasome inhibitor reduced muscle inflammation and partially restored dystrophin-associated glycoprotein complex. Bradykinin therapy improved heart function. Laminin-111 therapy enhanced regeneration and muscle repair. However, calpain inhibitor failed to ameliorate the dystrophic phenotype.

Cell therapy has been explored since the early ‘90s (177). A variety of cell types have been tested in the canine DMD model via intramuscular or intravascular delivery. These cell types include canine myoblasts (178, 179), canine mesoangioblast stem cells (180), human immature dental pulp stem cells (181), umbilical cord mesenchymal stromal cells (182), allogenic dog muscle stem cells isolated through serial plating (183, 184), human adipose-derived stromal cells (185, 186), and autologous engineered canine CD133+ cells (187). Collectively, these studies suggest that cell therapy is relatively safe and may achieve relatively long-term engraftment under certain circumstances. However, these studies also show that cell therapy for DMD has not reached its prime time yet.

The fundamental issue in DMD is dystrophin deficiency. Hence, the primary goal of DMD gene therapy is to restore dystrophin expression. Many approaches have been tried, including gene replacement with plasmid (e.g., Myodys), advenovirus, and AAV vectors, gene repair with CRISPR editing, and exon-skipping with antisense oligonucleotides (188–193). Gene replacement therapy has been tested using adenoviral- and AAV-mediated methods to deliver the dystrophin gene or engineered mini- or micro-dystrophin genes. With adenoviral gene therapy, relatively high-level dystrophin expression (~16%) was achieved under immune suppression in affected dogs following intramuscular injection of a first-generation adenoviral vector that expressed a 6.2 kb human mini-dystrophin gene (194). However, very few dystrophin positive cells were detected when the full-length human dystrophin cDNA was delivered using a high capacity adenovirus vector (195, 196).

Due to the smaller packaging capacity of AAV and the large size of the dystrophin gene, two approaches have been tried for AAV-mediated dystrophin replacement gene therapy. A highly truncated, micro-dystrophin gene (<4 kb) (197, 198) or dual-AAV vectors used in combination to deliver a larger payload (199, 200). The AAV micro-dystrophin vector has been evaluated by local, regional and systemic delivery in neonatal and young adult DMD dogs (201). AAV9 micro-dystrophin delivered by intramuscular injection in young adult DMD dogs showed reductions in muscle necrosis, inflammation, calcification, and fibrosis and prevented eccentric contraction-induced force drop (202, 203). This led to testing AAV8 and AAV9, where persistent body wide micro-dystrophin expression was detected up to 30 months post-injection without observable side effects. Micro-dystrophin restored the dystrophin-associated glycoprotein complex and mitigated inflammation, fibrosis, and calcification. Micro-dystrophin also improved growth, increased limb muscle force, and enhanced activity and gait. Collectively, preclinical studies in the canine model have provided compelling supporting data for conducting systemic AAV micro-dystrophin therapy in human patients (204–208). Three independent clinical trials (conducted by Solid Bioscience, Pfizer, and Sarepta) are currently ongoing (209).

Since many important protein interacting domains are missing in micro-dystrophin, delivery of a larger dystrophin gene by dual AAV vectors enabled packaging of a 10 kb genome (199, 200). A recent proof of principle study demonstrated efficient reconstitution of a split mini-dystrophin gene in the CXMD model by intramuscular injection, which greatly mitigated muscle pathology and improved contractility in the treated dog (210).

Only two canine studies have investigated the gene repair approach in the CXMD model (211, 212). Bartlett et al. injected a DNA/RNA chimeric oligonucleotide in the limb muscle of a 6-week-old GRMD puppy. They observed sustained correction of the point mutation in the genome and expression of the full-length dystrophin protein, but the efficiency was too low to mitigate muscle disease (211). CRISPR-mediated genome editing technology has significantly enhanced gene repair efficiency using AAV vectors encoding SpCas9 and gRNA to the ΔE50 canine DMD model (212). The ΔE50 dog contains a point mutation in the conserved splicing donor sequence of intron 50. This mutation results in erroneous skipping of exon 50 in the dystrophin transcript (213). Amoasii et al. designed a single gRNA that targets a conserved splicing enhancer sequence at the 5'-end of exon 51. Intramuscular or intravenous delivery of the gRNA and SpCas9 AAV vectors to 1-month-old ΔE50 puppies resulted in exon 51 re-framing or skipping in the transcript. Safe and robust dystrophin expression was detected for up to 2 months in most muscles in the body, including the heart. This pivotal proof-of-principle study provides critical support for continuous development of AAV CRISPR editing as a viable approach to treat DMD in human patients in the future (214, 215).

Canine DMD exon-skipping studies have been performed using antisense oligonucleotides (AONs) delivered either directly as a chemical/small molecule to the muscle or expressed from a U7snRNA AAV vector. Due to the unique location of the GRMD mutation, only multi-exon skipping (exons 6, 7 and 8) can result in an in-frame transcript. In the CXMDj dog, a beagle carrying the GRMD mutation was treated by three phosphorodiamidate morpholino oligomers (two targeting exons 6 and one targeting exon 8) by intramuscular and intravenous injection (216). Treatment resulted in widespread dystrophin expression in multiple skeletal muscles, attenuation of histological lesions and stabilization of clinical symptoms (216). Further optimized antisense oligonucleotide (AON) cocktail (e.g., vivo-morpholino and peptide-conjugated morpholino) showed near wild type level expression for at least 2 months following intramuscular injection and ~5% of the wild type level following intravascular injection in the heart and most skeletal muscles. Systemic delivery did not improve skeletal muscle histology and function. However, cardiac conduction abnormalities were significantly ameliorated (217, 218).

The AAV U7snRNA approach, where AAV encoded antisense oligonucletotides are fused with the U7 small nuclear RNA are transcribed from the U7 promoter (219–222), showed persistent dystrophin restoration, muscle force improvement and correction of the dystrophic phenotype. However, a gradual loss of dystrophin- positive myofibers was seen during a 5-year follow up (220, 222). Furthermore, the new study showed that limb perfusion of the U7snRNA AAV8 vectors did not induce the T cell response to newly synthesized dystrophin nor to AAV capsid (207). Cardiac rescue is attained with U7snRNA AAV6 vectors by percutaneous transendocardial delivery in the GRMD dog heart (219, 221). Collectively, these studies support initiation of a phase I trial in DMD patients.

An important concern of dystrophin gene replacement and repair therapy is the potential immune response to the novel dystrophin protein generated following the therapy. This concern can be greatly minimized if a therapeutic gene already exists in the patient's body. Utrophin, an autosomal paralogue of dystrophin, is highly enriched in the neuromuscular junction and myotendinous junction in normal muscle and is a potential dystrophin-independent strategy to circumvent the potential immune response to the novel dystrophin protein. An adenovirus carrying a 6 kb synthetic mini-utrophin gene was injected intramuscularly in newborn CXMD affected puppies and, in the presence of cyclosporin, mini-utrophin expression lasted for 2 months (223). More recently, an AAV vector carrying a micro-utrophin gene was tested in GRMD dogs and the German Shorthaired Pointer model (224, 225). In neonatal GRMD dogs, systemic AAV micro-utrophin delivery did not induce innate immune response. In the German Shorthaired Pointer model, the entire dystrophin gene is deleted. Intramuscular injection of an AAV micro-dystrophin vector to the adult German Shorthaired Pointer dogs induced the T cell response and had no muscle protection. However, injection of an AAV micro-utrophin vector resulted in robust micro-utrophin expression and muscle protection without inducing the T cell response (224).

An alternative approach to treat muscular dystrophy is to increase muscle mass by inhibiting the myostatin pathway. A secreted dominant-negative myostatin peptide with intravenous AAV injection in young adult GRMD dogs significantly increased muscle mass and reduced muscle fibrosis and the serum creatine kinase level (226). Interestingly, myostatin heterozygous GRMD dogs exhibited disproportional flexor muscle hypertrophy and atrophied extensor muscles (227). Subsequently, severe postural changes and aggravated joint contracture were observed.

A molecular hallmark of DMD is the aberrant elevation of the cytosolic calcium level. Calcium upregulation activates protease and phospholipase, triggers protein degradation and membrane damage, and eventually results in muscle necrosis; therefore, calcium homeostasis restoration has been suggested as a therapeutic target for DMD. Sarco/endoplasmic reticulum calcium ATPase 2a (SERCA2a) is a highly efficient calcium pump that transports cytosolic calcium to the sarcoplasmic reticulum. AAV-mediated SERCA2a expression has been shown to ameliorate muscle disease in dystrophic mice in young adult CXMD dogs (228, 229). Treatment significantly improved calcium uptake, reduced calpain activity and enhanced muscle function (230), suggesting that AAV SERCA2a delivery is a highly promising strategy to treat DMD.

## Mucopolysaccharidosis

### Mucopolysaccharidosis in Humans

In humans and animals, the mucopolysaccharide storage diseases (MPS) are due to lysosomal storage enzyme deficiencies leading to decreased degradation of glycosaminoglycans (GAGs). GAGs are important in cellular communication and regulation and provide structural support to the extracellular matrix; therefore, GAG accumulation occurs in many organ systems leading to a multisystemic clinical disease. Seven different forms of MPS (I-VII) exist due to defects in eleven enzymes (231). This review will focus on MPS I, III and VII.

*MPS I*, like most lysosomal storage diseases, is due to autosomal recessive inheritance leading to deficiency or absence of activity of α-L-iduronidase (IDUA) enzyme causing accumulation of heparan sulfate (HS) and dermatan sulfate (DS). MPS I was initially recognized as three clinical syndromes (Hurler, Hurler-Scheie, and Scheie) but due to clinical and biochemical overlap, a description of severe or attenuated is often implied. Common clinical signs in patients with MPS I include corneal clouding and subsequent vision loss, cognitive impairment, coarse facial features, hearing loss, macroglossia, cardiomyopathy, hydrocephaly, hepatosplenomegaly, hernias, and limited joint mobility (232). Patients with severe MPS I experience cardiorespiratory compromise and death within their first decade of life, while patients with the attenuated form have wide variation from death to a clinically normal life span (232).

*MPS III*, Sanfilippo syndrome, is due to autosomal recessive inheritance and is due to loss of function of one of the four enzymes involved in heparan sulfate catabolism leading to four different subtypes A-D. MPS III A and B are the most common forms seen clinically and most severe (233–237). The allelic heterogeneity and polymorphisms of the MPS III subtypes makes them difficult to separate based on phenotype. Interestingly, while other MPSs present with intense peripheral disease involvement, patients with MPS III typically present with neurologic and cognitive impairments (231). Although little is known about early disease development, MPS III mainly affects the neurons of the central nervous system causing dementia, mental retardation, delayed development, and neurological dysfunction (157, 238, 239). Typical neuropathological lesions include loss or ballooning of purkinje cells and accumulation of molecular layer dendrites with storage material (157, 240–242). Death usually occurs in the patient's early twenties to early thirties, for patients with the severe form, while patients suffering from the attenuated form have longer survival times (243–245).

*MPS VII*, caused by deficient activity of β-glucuronidase, was first described in a child in 1973 (246). This autosomal recessive disorder has multisystemic manifestations, including organomegaly and skeletal, central nervous system (CNS), cardiovascular, and ocular abnormalities (1). The prevalence at birth is reported to range between 1/345,000 and 1/5,000,000. However, the frequency of the disease may be underestimated as the most frequent presentation is the antenatal form, which remains underdiagnosed (231). MPS VII shares some symptoms with MPS I and MPS III, however, one unique and distinguishing clinical feature is the unexpectedly high proportion of patients (40–45%) with a history of non-immune hydrops fetalis (NIHF) (247). Cases are classified according to their phenotype into three different groups: (1) neonatal non-immune hydrops fetalis (NIHF), (2) infantile or adolescent form with history of hydrops fetalis and (3) infantile or adolescent form without known hydrops fetalis (248). The patients with prenatal-onset hydrops who survive gestation die in early infancy of heart, kidney or respiratory failure (247, 248). Patients surviving infancy can have a wide range of clinical manifestations from mild to severe; rare patients with milder manifestations of MPS VII have survived into the fifth decade of life (247, 248). Cognitive impairment is a common finding in MPS VII patients. Most patients have short stature, coarse facial features and skeletal abnormalities (231, 249). Almost half of patients with the infantile or adolescent form of the disease are able to walk by themselves without assistance; however, most patients lose this ability with time due to hip dysplasia by 10 years of age (247, 248). Corneal clouding, frequent upper respiratory infections, heart disease, hearing impairment and delayed speech development are among the clinical presentation of the disease (231, 247, 248). Common causes of death include complications of hydrops fetalis with respiratory and renal failure, heart disease, decreased pulmonary function and/ or obstructive airway disease and aspiration (247, 248).

### Mucopolysaccharidosis in Canines

#### MPS I

MPS I in canines most closely resembles MPS I Hurler syndrome in humans due to GAG and sphingolipid accumulations in CNS neurons in both species (250). Breeds typically affected include Plott Hounds, Miniature Pinchers, mixed-breed dogs (95), and Rottweilers (251). A multitude of clinical signs may be apparent due to GAG accumulation in many different organ tissues. Ocular changes and subsequent vision loss are common in canine MPS I. Corneal clouding occurs due to GAG accumulation in corneal stromal cells and scleral fibroblasts (252, 253). Aqueous flare, corneal neovascularization, and preiridal fibrovascular membranes are commonly observed (252–254). Vacuolation of bulbar conjunctiva, iris, choroid, and scleral cells is sometimes seen (253). Changes to the cardiovascular system include cardiac valvular insufficiencies, right ventricular enlargement, and arterial vascular lesions. In arteries, there is intimal thickening of the proximal and distal aorta with nodular plaques projecting from the walls behind the semilunar valves (255). Radiographically, joint effusion, lysis, severe secondary degenerative joint disease, periarticular bone proliferation, and narrowing of the intervertebral disc spaces may be evident (256). Corneal, dermal, and synovial biopsies show cells with various dilated vesicles and a fine granular internal matrix (256). On cerebral MRI, there is white matter volume loss of the corpus callosum, varying degrees of ventricular enlargement, cortical atrophy, and alterations in signal intensity (257).

#### MPS IIIa

Naturally occurring MPS IIIA has been described in Wirehaired Dachshund dogs and Huntaway dogs (258, 259). The onset of clinical symptoms in the Dachshund dog starts at 3 years of age and includes mild gait and postural reaction deficits in the thoracic limbs, coarse tremor of the head, pelvic limb ataxia and frequent stumbling and falling. The symptoms progress gradually over 2 years to severe generalized spino-cerebellar ataxia (258, 259). The Dachshund dogs contain a small amount of residual SGSH activity in skin fibroblasts. In contrast, Huntaway dog are completely deficient of SGSH activity, which may explain the severity of the disease in this canine model (259). The clinical onset of the disease in the Huntaway dog model occurs at 1.5 years of age with symptoms, including presence of ataxia and hypermetria in all four limbs, prancing gait affecting the forelegs, difficulty jumping, and loss of learned behavior (259). Huntaway dogs have similar disease progression and biochemical characteristics to that observed in severely affected MPS IIIA patients (259). The movement disorders appear in Huntaway dogs to be attributed to lesions in the cerebellum (260). Significant accumulation of secondarily stored lipids and subsequent loss of Purkinje cells is observed in this dog model (261). The loss of Purkinje cells and other abnormalities of the cerebellum are consistent with neurologic signs in Huntaway dogs, including ataxia, hypermetria, and a wide-based stance (261). Disease-related pathology is also severe in cerebral cortex and brainstem (261). In addition to accumulation of primary and secondary storage material, microgliosis and neuroinflammatory changes are present in the brain from the infant-pediatric-stage in MPS IIIA brain (262). In addition to brain, various tissues show disease related pathology (261).

#### MPS IIIb

MPS IIIb is observed in Schipperkes where the mutant allele is very prevalent (258, 259, 263). It is typically present in dogs around 2 years of age and leads to euthanasia prior to 5 years of age. Initial pathologic studies of Schipperkes showed that the major tissues affected in dogs (liver, kidney, CNS) were consistent with human. The most impressive finding in the Schipperkes with MPS IIIb was an almost complete loss of cerebellar Purkinje cells (263). Humans show varying degrees of cerebellar pathology leading to the belief that this may be a species sensitivity to HS accumulation seen in canines (263).

#### MPS VII

Canine MPS VII has been identified in mixed breed dogs, German Shepherds and Brazilian Terriers (264–266). Features in MPS VII dogs resemble those in humans, except mental retardation, which is difficult to assess, and hepatosplenomegaly which is less severe (267). Affected dogs excreted chondroitin 4- and 6-sulfates and dermatan sulfate in urine (268). The affected dogs present typical MPS VII features, including, facial dysmorphia, chest deformity, short stature, and corneal clouding (268). By 6 months of age effusions in knee and elbow joints are normally present and affected dogs are unable to stand or walk (267, 269). Cardiac abnormalities are variable between dogs and include mitral valve thickening and regurgitation, larger aortic dimensions in both the long and short axes, and aortic valves that are subjectively thicker (268, 270). Histologically, in the untreated dogs the vascular smooth muscle cells of the aorta become enlarged and rounded with highly vacuolated cytoplasm (268). In addition, prominent nodular thickening of the mitral valve is present with cytoplasmic vacuolation seen by light and electron microscopy, similar to that reported in children and to other animals with MPS (268).

### Therapeutic Testing in the Canine Model

There are different approaches available to treat MPS. Here we describe enzyme replacement therapy (ERT) and gene therapy (249, 269). ERT can be done through intravenous (IV) or intrathecal (IT) administration and internalization of the enzyme in cells occurs via the M6P receptor (M6PR), which present on the surface of most cells (271–273). IV ERT has been shown to be effective in treatment of canine model of MPS I by reducing lysosomal storage in liver, spleen, kidney, lymph nodes and lungs; however, the enzyme activity in brain is not significant (250, 274). IV ERT has been effectively used for treatment of non-neuronopathic MPS I, MPS II and MPS VI in different animal models; however, it is not effective for treating the neurological manifestation of MPS, as IV administered enzymes cannot be delivered beyond the blood-brain barrier (275). In addition, IV ERT does not address the disease pathology in joints and bone (276).

To address the limitations of IV ERT, IT ERT has been investigated, which resulted in improvements of clinical signs and/or neuropathology (277–280). IT administration of recombinant human iduronidase (rhIDU) in MPS I dogs, 4 times weekly, demonstrated that the enzyme can adequately penetrate the CNS, achieve levels that are many fold higher than normal and decrease GAG levels in brain, spinal cord and spinal meninges (281). Cisterna magna administration of rhIDU, repeated weekly, monthly or quarterly, in canine MPS I showed promising results where the mean iduronidase levels in brain, spinal cord and spinal meninges were several folds above normal and GAG storage in the brain was normalized (282). In Huntaway dogs, weekly injection of recombinant human sulfamidase (rhSGSH) into the cerebellomedullary cistern for up to 4 weeks, resulted in dose dependent distribution of SGSH to the brain and spinal cord. At high concentrations, rhSGSH was detected in cerebellum, brainstem, all levels of the spinal cord and most rostral areas of the cerebral cortex; however, deep brain structures, e.g., hippocampus, caudate and thalamus, showed lower rhSGSH activity (283). This study was repeated with treatment of younger MPS IIIA dogs (up to 23 weeks of age) and demonstrated that the enzyme delivery via CSF reduces neuropathology (284).

To overcome the limitations of ERT, *ex vivo* and *in vivo* gene therapies have been investigated in small and large animal models of MPS. Hepatic gene therapy in MPS VII dogs using retroviral vector expressing canine beta-glucuronidase GUSB resulted in normal activity of serum GUSB for up to 14 months (267). Treated MPS VII dogs had improvements in bone and joints abnormalities and retained the ability to run. They did not develop valve thickening and had no corneal clouding (267). Neonatal retroviral gene therapy in MPS VII dogs has been shown to have long term effects and could increase the life span of treated dogs up to 11 years, with treated MPS VII dogs retaining the ability to walk during their extended lives and showing reduced cardiac valve disease (285).

AAV gene therapy has also been used for treatment of MPS VII dogs. Safety and efficacy of AAV5, directly injected into putamen and centrum semiovalae, was demonstrated in MPS I and MPS IIIB dogs (286). Cisterna magna injection of AAV9 expressing human IDUA in MPS I dogs resulted in widespread CNS transduction and subsequent correction of brain storage lesions in a dose-dependent manner (287). The thickening of meninges, which contribute to spinal cord compression in MPS I patients was reversed (287). Another study compared therapeutic effect of IV, cisterna magna and combined routes of injection of AAV9 or AAVrh10 vectors expressing canine GUSB in MPS VII dogs (288). While IV treated dogs showed reduced storage in non-CNS tissues and low GUSB expression in CNS, the IT treated dogs had widespread GUSB activity in neuronal tissues, including cerebrum, spinal cord and sciatic nerve. The dogs that received the dual treatment using two different AAV serotypes maintained mobility throughout the study. The GUSB levels in CNS of this group mimicked the IT only dogs and the peripheral tissue levels mirrored those of IV treated dogs (288).

### Impact on Patients With This Disease

Enzyme replacement therapy (ERT) via IV infusion of recombinant human enzyme is available for MPS I, II, and VI in the United States, Europe, and over 40 countries worldwide (289). IV ERT received FDA approval for MPS VII treatment in 2017. Studies of patients during the trial and follow-up studies indicate that this treatment can stabilize or reverse many aspects of the disease. IV ERT has been shown to decrease the urinary GAG excretion, size of liver and spleen, progression to heart failure and sleep apnea for MPS I patients (290–292). Overall, patients have improved ability to perform normal activities of daily living. However, cardiac valve disease and skeletal disease do not generally get treated with ERT if pathological changes are already present (276, 289). Airway disease improves, but usually does not normalize (289). These observations suggest that early initiation of therapy before the onset of clinical symptoms may be more beneficial (289). Clinical observation has not supported neurocognitive benefit for patients with CNS manifestation since the enzyme at the labeled doses cannot cross the blood-brain barrier (289, 293). IT ERT has been performed to address the CNS disease of MPS IIIA patients. A phase I/II study using an IT drug delivery device demonstrated the safety of this delivery method, and a consistent decline of the heparan sulfate in CSF was documented in all patients following the administration of the first dose (294).

AAV gene therapy as a strategy to treat systemic or CNS manifestation of MPS is being investigated in ongoing clinical trials for different types of MPS, including I, IIIA, and IIIB using AAV6, AAV9, and AAVrh10 (clinicaltrials.gov: NCT02702115, NCT03580083, NCT03315182, NCT04088734, NCT02716246, NCT03612869).

## Fucosidosis

### Fucosidosis in Humans

Fucosidosis is a rare, autosomal recessive, lysosomal storage disease caused by a deficiency of α-L-fucosidase. The biological role of this enzyme is to catalyze removal of fucose moieties from glycoproteins and glycolipids, therefore it affects all tissues. Enzymatic deficiency leads to accumulation of storage material composed mainly of glycoasparginases (glycoproteins) with mucopolysaccharides, oligosaccharides, and glycolipids accumulating to lesser extents (295–298). Storage accumulation takes place in the skin, heart, pancreas, liver, thymus, kidneys, and brain. Accumulation of glycolipids and glycoproteins in the brain leads to cell death and neurologic symptoms. Fucosidosis is progressive, resulting in cognitive and progressive motor impairments as the most characteristic clinical signs. Common clinical signs in human patients include mental retardation, neurologic deterioration, coarse facial features, growth retardation, recurrent infections, dysostosis multiplex, angiokeratoma, seizures, and organomegaly (296). A combination of dark and clear vacuoles is unique and typical for fucosidosis with cellular vacuolation of the liver, brain, peripheral nerves, pancreas, skin, conjunctiva, and fibroblasts present to varying degrees on histopathology (296). Infantile cases have been shown to exhibit early CNS myelin loss as a pathological feature (299–302). The mutation causing enzymatic deficiency resides in FUCA 1 gene (303) with early analyses describing obliteration of an Eco RI restriction site and deletions of at least two exons at the 3'-end of FUCA1 (296).

Fucosidosis has a worldwide distribution and has been classically identified as taking two forms: a severe form, type I, and a less severe form, type II with further investigations pointing toward a clinical continuum rather than distinct disease forms. A diagnosis is confirmed by α-L-fucosidase activity levels below 5% in the urine, leukocytes, cultured fibroblasts, or other tissues obtained by biopsy or autopsy (296). Fucosidosis displays a bimodal distribution of survival in human patients (296) with 43% dying before 10 years of age and 41% after age twenty, and patients with early symptom presentation and rapidly progressive disease tending to have shorter lifespans.

### Fucosidosis in Canines

As in humans, fucosidosis in canines is due to an autosomal recessive enzymatic deficiency of α-L-fucosidase (304) and is the only animal model for the human disease. In dogs, fucosidosis affects English Springer Spaniels and is a severe, progressively fatal neurodegenerative disease (305). Compared to humans, canines show a relative consistency in neurologic clinical signs and pathology. Neurologic signs typically present around 12–18 months and progress to death or euthanasia before the animal reaches 4 years of age. In the first year of life affected animals are clinically normal. Gait and behavior deficits (resistance to restraint, anxiety) manifest prior to 30 months of age progressing to severe ataxia and mental deterioration that requires humane euthanasia prior to 40 months of age (306). Canine patients may exhibit various neurologic abnormalities, such as postural reaction deficits, muscle tremors, circling, nystagmus, dysphagia, regurgitation, falling, unpredictable behavior, and jaw chattering (307). The molecular defect underlying canine fucosidosis is due to a 14 base pair deletion at the 3' portion of exon 1 resulting in a frameshift and subsequent production of a premature stop codon in exon 2, producing a foreshortened and unstable protein product (308).

The frontal cerebral cortex of affected dogs shows extensive cytoplasmic vacuolation of neurons and glial cells (305). The cerebellum, striatum, thalamus, and corpus callosum show intermediate pathology and there is an absence of vacuolation in the lumbar spinal cord (309). This excessive vacuolation can lead to palpably enlarged peripheral nerves on physical examination as well as vacuolated lymphocytes on blood smears and bone marrow samples (304). Hypomyelination is a characteristic of many lysosomal storage diseases and is present in human and canine fucosidosis. In dogs, the survival of the oligodendrocytes in the early myelinating cerebellum is significantly reduced due to excessive vacuolation, increased oligodendrocyte apoptosis leading to reduced expression of myelin-related genes and decreased axonal density (307). Definitive diagnosis is reached by demonstrating reduced α-L-fucosidase in leukocytes, plasma, or skin fibroblasts and should be suspected in young adult English Springer Spaniels with neurological deterioration. A diagnosis is further supported by palpably enlarged peripheral nerves, blood smear lymphocytic vacuolation, or vacuolation in peripheral nerve biopsies (304).

### Therapeutic Testing in the Canine Model

Bone marrow transplantation (BMT) and intracisternal enzyme replacement therapy (ERT) are the two current treatment modalities for canine fucosidosis. BMT was first explored in the early 1990s and remains the treatment of choice to amend the clinical disease associated with fucosidosis (310). Animals that maintained long term engraftment and were treated at 2–4 months of age, before onset of clinical signs, showed definitive reduction of neurologic disease and improvement in pathologic lesions. Whereas, animals that were treated after 6 months of age, late in clinical disease, showed little improvement in clinical signs and central neuronal storage. These findings illustrate that BMT in severely affected human patients is not recommended. After BMT, all dogs showed a rapid and sustained increase in α-L-fucosidase activity in plasma and leukocytes with stabilization at over 100% of donor levels in leukocytes and 30% of donor activity in plasma (310). Unfortunately, BMT is not benign and includes substantial associated risks, need for appropriate donors, and limited use for severely affected patients.

Low dose monthly intracisternal infusions of canine recombinant α-L-fucosidase in patients with fucosidosis resulted in increased enzymatic activity by 2–72% in the spinal cord and most areas of the brain with substrate reduction ranging from 0 to 80% (311). ERT showed significant improvements in cortical astrocytosis, LAMP-1 gene expression, deep cerebrocortical vacuolation, and superficial cortex ‘vacuole per neuron' while showing small improvements in microgliosis, myelination, and cortical neuron loss (312). Studies of ERT in canine fucosidosis have shown increased survival of cortical pyramidal neurons with little effect on cerebellar neurons demonstrating that cerebellar clinical signs (ataxia, hypermetria, proprioceptive deficits) may not be prevented (312). Additionally, the enzymatic activity in patients with ERT proved unsatisfactory in reducing substrate levels in many CNS regions and did not match the sustained enzymatic activity achieved with BMT (311).

### Impacts on Patient With This Disease

BMT has been carried out in human patients with results similar to canine patients (302, 313). As previously mentioned, dogs are the only large animal model for this disease and studies in fucosidosis dogs have helped pave a path for BMT in humans. Accurate diagnosis must be made early in disease pathogenesis as CNS deterioration progresses to death and may not be corrected with later transplatation. Long term follow-up in canine and human patients is required to better evaluate the benefits of BMT in regard to quality of life and progression of neuropathology, although, until gene therapies become readily available, BMT remains the treatment option for an otherwise lethal disease.

## Summary

Dogs faithfully recapitulate human diseases, with similar phenotypes, biodistribution complexity, immune responses and therapeutic efficacy as human patients. These models have proven invaluable in the development of the therapeutics described here and testing in these models continues to result in promising results, worthy of first-in-human clinical trials for these terrible diseases.

## Author Contributions

BS wrote the abstract, introduction, and the sections Alzheimer's disease in humans, Canine cognitive dysfunction, amyotrophic lateral sclerosis, degenerative myelopathy for degenerative myelopathy, neuronal ceroid lipofuscinosis in humans, neuronal ceroid lipofuscinosis in canines, globoid cell leukodystrophy in humans, globoid cell leukodystrophy in canines, duchenne muscular dystrophy in humans, canine X-liked muscular dystrophy, and fucosidosis. MM wrote the sections Therapeutic testing in the canine model, impacts on patient with disease for canine cognitive dysfunction, therapeutic testing in the canine model, and impacts on patients with this disease for degenerative myelopathy. AB wrote and edited therapeutic testing in the canine model, and impacts on patients with this disease for globoid cell leukodystrophy. SB wrote therapeutic testing in the canine model and impacts on patients with this disease for neuronal ceroid lipofuscinosis. DD and EM wrote and edited the section Therapeutic testing in the canine model. DD edited the sections Duchenne muscular dystrophy in humans and canine X-linked muscular dystrophy. DFa contributed to amyotrophic lateral sclerosis by providing background, experimental details of gene therapy clinical trial in degenerative myelopathy dogs. DFa also provided extensive unpublished data, which was beyond the scope of this manuscript but helpful in shaping the section. TT wrote MPS VII in mucopolysaccharidosis in humans and MPS IIa and MPS VII in mucopolysaccharidosis in canines and therapeutic testing in the canine model and impacts on patients with this disease for mucopolysaccharidosis. DFe assisted in the compilation and editing of the manuscript. HG-E oversaw manuscript preparation, collation of sections, drafting and final editing.

### Conflict of Interest

DD is a member of the scientific advisory board for Solid Biosciences and an equity holder of Solid Biosciences. The Duan lab has received research supports unrelated to CRISPR editing from Solid Biosciences and Edgewise Therapeutics. The remaining authors declare that the research was conducted in the absence of any commercial or financial relationships that could be construed as a potential conflict of interest.
